# Pandemics of the 21st Century: The Risk Factor for Obese People

**DOI:** 10.3390/v14010025

**Published:** 2021-12-23

**Authors:** Miriam Hancková, Tatiana Betáková

**Affiliations:** 1 Biomedical Research Center, Slovak Academy of Sciences, Institute of Virology, 845 05 Bratislava, Slovakia; virumiha@savba.sk; 2 Department of Microbiology and Virology, Faculty of Natural Sciences, Comenius University, 842 15 Bratislava, Slovakia

**Keywords:** obesity, SARS-CoV-2, influenza, immune system, vaccination

## Abstract

The number of obese adults and children is increasing worldwide, with obesity now being a global epidemic. Around 2.8 million people die annually from clinical overweight or obesity. Obesity is associated with numerous comorbid conditions including hypertension, cardiovascular disease, type 2 diabetes, hypercholesterolemia, hypertriglyceridemia, nonalcoholic fatty liver disease, and cancer, and even the development of severe disease after infection with viruses. Over the past twenty years, a number of new viruses has emerged and entered the human population. Moreover, influenza (H1N1)pdm09 virus and severe acute respiratory syndrome coronavirus 2 (SARS-CoV-2) have caused pandemics. During pandemics, the number of obese patients presents challenging and complex issues in medical and surgical intensive care units. Morbidity amongst obese individuals is directly proportional to body mass index. In this review, we describe the impact of obesity on the immune system, adult mortality, and immune response after infection with pandemic influenza virus and SARS-CoV-2. Finally, we address the effect of obesity on vaccination.

## 1. Introduction

Changes in host population ecology, genetic mutations in pathogen reservoirs, the lack of disease control, the growing population, overcrowded urban areas, climate change, and globalization have resulted in the development of several epidemics and two pandemics in the 20th and 21st centuries. Due to the advances in medical science, raising awareness of the risk factors, educating people, and adopting safety measures, the severe acute respiratory syndrome (SARS) outbreak in China (2002), avian influenza virus H5N1 outbreaks (2005), Zimbabwean Cholera outbreak (2008), West African meningitis outbreak (2009), Haitian cholera outbreak (2010), dengue fever outbreak in Pakistan (2011), yellow fever outbreak in Sudan (2012), Middle East respiratory syndrome (MERS) outbreak (2012), West African Ebola virus epidemic (2013), zika virus epidemic in Brazil (2015–2016), Yemen cholera outbreak (2016), and Nipah virus outbreak in India (2018) did not spread worldwide or cause pandemics. Nevertheless, two pandemics originated in the past 100 years. The new influenza virus A(H1N1)pdm09 virus emerged in Mexico and became an epidemic in 2009. This H1N1 strain of influenza virus had not circulated previously in the human population and was entirely new [1]. The virus was contagious, spread easily from one person to another, and replaced the seasonal H1N1 virus, which was, by then, circulating in the population. The novel (H1N1)pdm09 virus preferentially infected younger people and most cases of severe and fatal infection appeared in adults aged between 30 and 50 years [2]. The researchers estimated that between 123,000 and 203,000 people younger than 65 years old died from influenza in 2009 [3]. Each year, 3 to 5 million individuals are severely affected by the influenza virus and 250,000 to 500,000 individuals do not survive this infection [4]. Globally, coronavirus disease 2019 (COVID-19) was declared a pandemic in March 2020 by the World Health Organization (WHO). COVID-19 is caused by infection with severe acute respiratory syndrome coronavirus 2 (SARS-CoV-2) has about one hundred different symptoms. To date, more than 240 million people have experienced infection with SARS-CoV-2, of which nearly 5 million people have died.

Influenza and SARS-CoV-2 are intracellular respiratory pathogens that are highly infectious. Several factors have been identified as a health risk, such as age (children younger than 5 years and people older than 65 years), chronic comorbidities, pregnancy, undernutrition or overnutrition, obesity, and cardiovascular or oncological disease. These risk factors may be directly linked to poor prognosis of infection, associated with increased morbidity and mortality [5]. Evidence from many studies indicates that excess weight and obesity are associated with the development of severe infections in the respiratory tract in these patients, and hospitalization compared to nonobese individuals [6,7,8]. These individuals are more susceptible to various intracellular pathogens, mainly to viruses, because their immune systems are suppressed and they more frequently experience some physiological, immune, or metabolic changes [9,10].

## 2. Influenza A Virus

According to international taxonomy, the clinically important genus Influenza A virus is classified into the family Orthomyxoviridae. This group is characterized by single-stranded RNA viruses with negative sense. The viral genome is segmented and codes different structural and nonstructural proteins that are important in viral replication and pathogenesis in influenza infection. The total genome size is approximately 13.6 kilobase pairs (kb) in length [11]. The morphological structure of influenza infectious particles is predominantly roughly spherical and enveloped with a lipid bilayer membrane that is removed from the host cell. The lipid membrane includes two major surface glycoproteins, hemagglutinin (HA) and neuraminidase (NA), and one integral viroporin, the protein M2. The core of influenza virions is formed of eight RNA segments that are encapsulated with nucleoprotein (NP) and associated with a heterotrimeric RNA-dependent RNA polymerase complex that consists of polymerase proteins (PB1 and PB2) and an acidic protein (PA). The nucleoprotein protects the viral RNA against damage and forms a loop at one end. Together, the above-mentioned components form the viral ribonucleoprotein complex (vRNP) [12,13]. In addition, the viral genome of influenza A virus encodes nine noncoding proteins [14,15].

## 3. Virus SARS-CoV-2

SARS-CoV-2 belongs to the family Coronaviridae and genus Betacoronavirus [16]. This novel type of coronavirus is an enveloped single-stranded RNA virus with positive-sense that infects respiratory epithelial cells and has contributed to the development of pandemic pneumonia [16,17]. Viral particles are typically round and spherical in shape. The genome size is approximately 29.9 kb. It exhibits about 79.5% homology to the native strain of SARS-CoV and about 50% homology to the Middle East respiratory syndrome coronavirus [18,19,20]. The viral genome is nonsegmented. The molecular structure of the viral particle of SARS-CoV-2 consists of five structural proteins: nucleocapsid protein (N), membrane glycoprotein (M), spike glycoprotein (S), hemagglutinin-esterase (HE), and an envelope protein (E). The viral S, M, HE, and E proteins are integrated into the lipid bilayer, which is derived from the host cell membrane. The structural N protein envelopes and is directly associated with the viral RNA in the core of the virion. In addition, the SARS-CoV-2 genome codes sixteen nonstructural proteins (nsp1–nsp16) that play a significant role in the pathogenesis of COVID-19 [16,21,22].

## 4. Obesity and Its Comorbid Conditions

The body fat percentage is expressed as body mass index (BMI), which is commonly used to classify underweight, overweight, or obese in adults and children. The body mass index is calculated as the ratio of body weight to body height, measured in kilograms (kg) and square meters (m^2^), respectively [23]. A BMI over 25 is considered overweight, and over 30 is obese; these values are the same for both sexes and for all ages of adults. The definition of overweight or obese depends on age in the child population. In 2016, it was estimated that more than 1.9 billion adults (≥18 years of age) worldwide were overweight, and over 650 million were obese. Excess weight was recorded in 39% of men and 40% of women in the adult population; additionally, 11% of men and 15% of women were obese. Over 340 million children (5–18 years) suffered with excess weight and obesity in 2016 [24]. According to statistics from 2019, the prevalence of excess weight and obesity in children has increased significantly, with an estimated 40 million patients who were under 5 years of age [25]. It was predicted that nearly 50% of the world population will become obese by 2050.

Nowadays, overweight and obesity kill more people in developed countries than being underweight in developing countries. At least 2.8 to 4 million deaths per year are recorded as resulting from incipient excess weight and morbid obesity [26]. Overweight and/or obesity is a chronic disease characterized as an abnormal and excessive amount of body fat that can impair the immune system [27,28]. Obesity may present a serious health risk factor, being is often linked to the development of chronic inflammatory diseases, e.g., type-2 diabetes, hypertension, cardiovascular diseases, chronic lung diseases, and cancer [29,30,31]. These comorbidities are associated with a severe clinical course of respiratory infection caused by influenza virus and SARS-CoV-2 infection. As a serious health risk factor, obesity leads to more frequent hospitalization and increasing morbidity and mortality worldwide compared to nonobese individuals [23]. Child obesity is mainly associated with a worse course of respiratory diseases [27].

## 5. Impact of Obesity on the Immune System

An obese state is associated with various physiological and anatomical changes within the body that may contribute to impaired immunity and modulation of antiviral immune responses. These changes occur mainly in the respiratory system, in which the volume of the lung is markedly decreased, which is related to increased respiratory rates and the development of shortness of breath [32,33,34]. Obesity is identified as an overaccumulation of white adipose tissue, which results in the overexpression of adipokines, also known as cytokine-like hormones. This may be a mechanism that contributes to the inflammatory and immunocompromised state during obesity [9,32,35].

**Figure 1:**
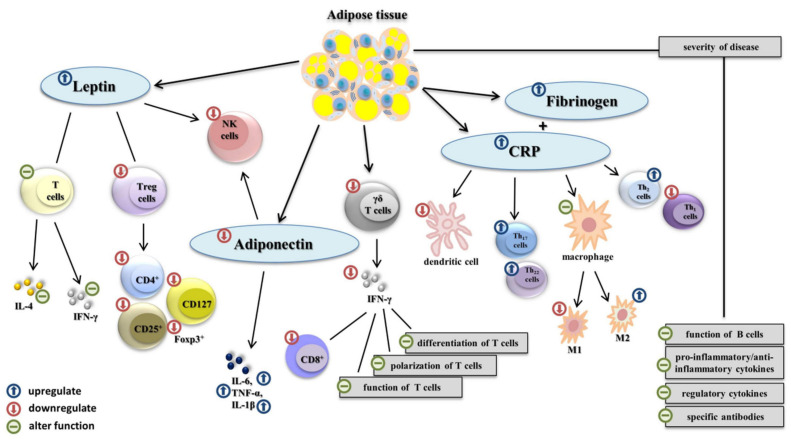
The impact of obesity on the immune system. In obese individuals, overaccumulated white adipose tissue overexpress adipokines such as leptin adiponectin, C-reactive protein (CRP), and fibrinogen. The leptin receptor is expressed on the surface of T lymphocytes and alters the function of pro-inflammatory IFN-γ and IL-4. Hyperleptinemia is associated with decreased activity of NK cells and lower level of Treg, which lead to a lower amount of sufficient CD4^+^, CD25^+^, and CD127 markers and Foxp3^+^ transcription factor. A decreased concentration of adiponectin alters the function of NK cells. Pro-inflammatory cytokines TNF-α, IL-1β, IL-6, and CCL2 are constantly overexpressed. Lower levels of γδ T cells lead to a reduced production of pro-inflammatory IFN-γ, and alter the adaptive immunity response. Overexpression of CRP impairs the homeostasis between Th1 and Th2 cell subsets, increases the activity of Th17 and Th22, decreases the level of M1 macrophages (M1), increases the level of M2 macrophages (M2), and decreases the number of dendritic cells. Obesity negatively influences the number of B cells, and the production and secretion of pro-inflammatory, anti-inflammatory cytokines, regulatory cytokines, and specific antibodies in infected individuals, which negatively affect disease severity.

Adipokines are proteins secreted by adipose tissue and play a crucial role in communication with other organs and tissues. Similarly, adipokines influence a wide range of physiological functions within the body, e.g., metabolic pathways, inflammatory processes, and immune responses (Figure 1). The necessary and relevant adipokines include leptin, adiponectin, and/or the pro-inflammatory cytokines TNF-α, IL-1β, IL-6, and CCL2 [35,36,37]. Leptin is a nonglycosylated protein (as a pro-inflammatory adipokine) that is directly linked to obesity and outcomes such as hyperleptinemia and participates markedly in the stimulation and activation of the JAK/STAT intracellular signaling pathway as part of the immune response [38,39,40]. Leptin receptors are expressed on the surface of T lymphocytes and are related to increased secretion of pro-inflammatory IFN-γ and reduced production of pleiotropic IL-4 in nonobese adult and pediatric patients [40]. However, hyperleptinemia is associated with decreased activity of NK cells, which may be connected to leptin resistance [9]. In contrast, the concentration of adiponectin (which is an anti-inflammatory adipokine) is significantly lower in obese individuals. This has been linked to an altered cytotoxic function of NK cells and the production or secretion of cytokines by effector myeloid cells. Pro-inflammatory cytokines such as TNF-α, IL-1β, and IL-6 are constantly overexpressed during obesity, which leads to the impairment or complete failure of the innate immune response [35,37,41]. Furthermore, increased expressions of C-reactive protein (CRP) and fibrinogen are closely related to an obese state [42,43].

The outcomes of clinical studies suggest impaired antigen presentation is due to a reduced function of macrophages and a decreased number of dendritic cells (DCs) in obese individuals. Clinical experiments also suggest that levels of phenotype M1 macrophages are considerably lower than levels of M2 macrophages in these individuals [42,44,45]. Circulating γδ T cells are decreased in obese individuals. These cells play a key role in the epidermal barrier. Lower levels of γδ T cells lead to a reduced production of pro-inflammatory IFN-γ in antiviral responses [42,46].

Overweight and obesity are also strongly associated with an adaptive immune response. In obese adult and pediatric patients, there was a significant change in the differentiation, polarization, and function of T-cell subsets, especially the amount of cytotoxic CD8^+^ T lymphocytes [32,47]. The physiological homeostasis between Th1 and Th2 cell subsets shifts to the population of helper Th2 lymphocytes during the antiviral immune response in obese individuals. Subsets of Th2 cells were correlated with the expression of C-reactive protein in peripheral blood. Similarly, levels of circulating Th17 and Th22 cells can also be higher [48,49,50,51]. Notably, obese individuals exhibit lower levels of regulatory T cells (Treg), which play a central role in the control of immune tolerance and directly correlate with the BMI index and levels of leptin. This decreased concentration of Treg cells is linked to a lower amount of sufficient CD4^+^, CD25^+^, and CD127 markers, and Foxp3^+^ transcription factor [52,53]. In addition, humoral immunity is affected by adipokines in obese individuals. The overexpression of adipokines negatively correlates with the secreted amount and function of B cells. Decreased differentiation and polarization may lead to altered secretion and the production of pro-inflammatory, anti-inflammatory, or regulatory cytokines, and the release of specific antibodies in obese patients [54,55,56].

Obesity is also associated with impaired glucose homeostasis. The increased plasma glucose and circulating lipids have a deleterious connection to glucose metabolism. Lipotoxicity is responsible for functional impairments in several metabolic pathways not only in adipose tissue, but also in the pancreas, liver, heart, and muscle. Increased level of lipids circulating in the blood as well as the intracellular signaling and metabolic alterations in fatty acid employment are related to insulin resistance [57,58]. The increased numbers of macrophages, CD8^+^ effector/effector memory T cells, Th1 cells, and chronical inflammation are also associated with insulin resistance. In addition, cells of the immune system such as NK cells, neutrophils, type 1 innate lymphoid cells (ILC1 cells), and B2 cells contribute to the development of insulin resistance in obese individuals [31].

In summary, the overexpression of adipokines exhibits negative effects in terms of the pathogenesis and severity of infectious diseases

## 6. Clinical Data in Obese Patients with Influenza and COVID-19

**Figure 2:**
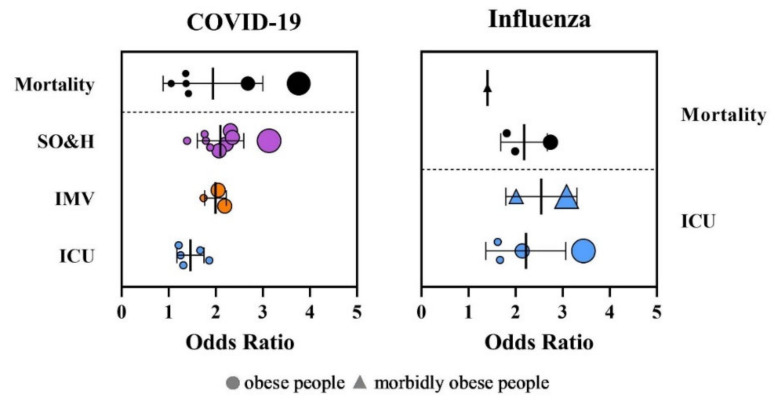
Distribution of odds ratios (OR) for intensive care unit administration (ICU), invasive mechanical ventilation (IMV), severe outcome and hospitalization (SO&H), and mortality in patients with obesity after infection with SARS-CoV-2 and influenza virus. The size of the dots corresponds to number of analyzed patients.

The association between increased BMI and severity of virus infections has been reported in all parts of the world. We compared the effects of obesity on disease severity and hospitalization, intensive care unit administration (ICU), invasive mechanical ventilation (IMV), and mortality of both influenza and COVID-19 based on the available evidence from systematic reviews and meta-analyses (Figure 2, Tables S1 and S2). The data show significant heterogeneity among the regions in Europe, Asia, and the Americas in COVID-19 studies, indicating the existence of specific regional characteristics of COVID-19, which might cause different BMI definitions and criteria for admission to ICUs and the need for IMV.

The meta-analyses showed that obese patients with a BMI ≥ 30 kg/m^2^ had an increased risk of severe outcome and hospitalization due to COVID-19 (odds ratio (OR) = 1.39–3.13) [59,60,61,62,63,64,65,66,67]. Obesity was also associated with a higher risk of ICU admission (OR = 1.21–1.8) and higher risk for IMV (OR = 1.74–2.19) [60,61,62,64,68]. The patients with higher BMI required IMV more frequently and for longer. The impact of obesity on the mortality of COVID-19 is not explicit. While some studies did not prove an increased risk on mortality due to obesity [59,61,65,66,68], other studies clearly linked obesity with increased mortality risk (OR = 1.05–2.68) [60,62,63,67,69]. Explicit data regarding morbid obesity with a BMI ≤ 35.0 kg/m^2^ are lacking, although studies have reported a clear association between morbid obesity and a highly increased risk of mortality (OR = 3.76; 95% CI: 2.67–5.28) [63].

The obesity pandemic appeared in the 20th and 21st centuries; therefore, evidence of the obesity risk factor for the development of complicated or severe influenza illness and mortality is missing for the pandemics that occurred in 1918, 1957, and 1968. Limited information is available from the influenza epidemic in 2009 (Figure 1, Table S2). More than 50% of A(H1N1)pdm09 virus patients had a BMI ≥ 30 kg/m^2^ [5,70,71,72,73,74,75,76]. The risk of hospitalization for respiratory illness during seasonal influenza is 1.45 times higher for obese adults with a BMI ranging from 30.0 to 34.9 kg/m^2^, and 2.12 times higher for severely obese adults with a BMI ≤ 35.0 kg/m^2^ [77]. A meta-analysis that included the latest studies from different countries showed that obesity is linked with a higher risk for intensive care unit administration and mortality with pandemic influenza with OR = 1.56–3.44 for a severe outcome and OR = 1.81–2.74 for mortality. Patients with morbid obesity have an extremely higher risk of being admitted to hospital (OR = 2.01–3.08) and death (OR = 1.40) [63,78,79,80].

## 7. Course of Influenza and COVID-19 in Obese Patients

Disruption of the immune response and dysfunction of the metabolic system responsible for chronic and low-grade inflammation are associated with a worse course of respiratory infections, increased mortality, and development of different comorbidities. Adipose tissue is plentifully populated by myeloid and lymphoid cells, and obesity is associated with lymphocyte and macrophage dysfunction [81,82]. As mentioned above, specific inflammatory cytokines such as TNF-α, IL-1β, and IL-6 are permanently induced in obese people; thus, viral infection merely boosts the signal for a cytokine response in already primed adipose tissue. Moreover, the sustained activation of multiple cytokine pathways by IL-6 plays a major role in the development of cytokine storm and in the pathogenesis of virus infection [83]. Obese people are more susceptible to virus infection and have worse prognoses due to associated viruses from secondary infection, co-infection, and opportunistic infection.

**Figure 3:**
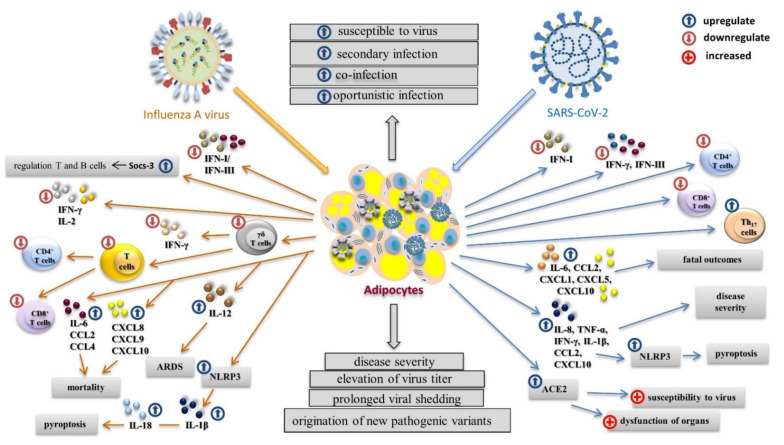
The impact of virus infection on the immune response in obese individuals. With influenza infection, the antiviral response and immune disfunction of adaptive immunity are inefficient due to the chronic production of pro-inflammatory cytokines. The production of IFNs I and IFNs III is delayed or increased, and blood peptin upregulates the suppressor of cytokine signaling-3 (SOCS-3), which regulates the function of T and B cells. The reduced amount and activity of γδ T cells leads to a lower production of IFN-γ and reduced T cell diversity. The influenza virus activates assembly of the pyrin domain-containing protein 3 (NLRP3) inflammasome, which supports the autocatalytic processing of pro-caspase-1 and the subsequent cleavage and secretion of pro-inflammatory cytokines IL-1β and IL-18, leading to pyroptosis. The development of cytokine storm results in the overexpression of IL-6 and chemokines CCL2, CCL4, CXCL8, CXCL9, and CXCL10. With SARS-CoV-2 infection, the production of IFN-β and IFN-λ is attenuated, and the level of IFN-γ is very low. Concentrations of CD4^+^ and CD8^+^ T lymphocytes are reduced and the level of Th17 is increased. SARS-CoV-2 upregulates the expression of five cytokines connected to mortality: IL-6, CCL2, CXCL1, CXCL5, and CXCL10. Activation of the NLRP3 inflammasome with increased levels of TNF-α, IFN-γ, IL-1β, IL-8, CCL2, and CXCL10 results in pyroptosis. Overexpression of the ACE receptor plays a crucial role in increased susceptibility to SARS-CoV-2, higher infectivity, and the dysfunction of organs. Both influenza virus and SARS-CoV-2 can infect adipose tissue, which can serve as a virus reservoir, prolonging viral shedding and supporting the origination of new pathogenic variants.

Influenza virus infection triggers the expression of interferons (IFNs), the key immune regulators against viral infection (Figure 3). Interferons I (IFN-α, IFN-β, and IFN-ε) and interferons III (IFN-λ1–IFN-λ4) control influenza virus replication [84,85]. However, influenza viruses have developed multiple strategies to regulate the host immune response and cellular signaling pathways. Delayed or a considerably increased production of IFN I can increase lethality during some acute influenza virus infections. Obese individuals have an increased level of blood peptin, which upregulates the suppressor of cytokine signaling-3 (SOCS-3). SOCS-3 regulates the IFN response as well as immune function of T and B cells. IFNs I regulate the antiviral function of γδ T cells [86,87]. The reduced concentration and activity of γδ T cells lead to a lower production of IFN-γ and reduced T cell diversity, which result in a poor response to influenza infection [46]. The attenuated and prolonged IFN response and pro-inflammatory cytokines in obese individuals lead to antiviral inefficacy and immune disfunction of adaptive immunity [88]. It is well-known that higher BMI values are associated with a reduction in CD4^+^ and CD8^+^ T-cell activation and function in post-influenza A(H1N1)pdm09 infection compared to uninfected lean individuals. The influenza virus also activates the assembly of the pyrin domain-containing protein 3 (NLRP3) inflammasome in hematopoietic cells as well as in some nonimmune cells such as primary bronchial epithelial cells, lung fibroblasts, and various epithelial cell lines [89,90,91]. The assembly of NLRP3 supports the autocatalytic processing of pro-caspase-1 and the subsequent cleavage and secretion of pro-inflammatory cytokines IL-1β and IL-18. The NLRP3 inflammasome plays a critical role in mediating antiviral immune responses during influenza virus infection [89,90,92]. However, NLRP3 is also activated in the adipose tissue of obese individuals, in which the assembly of Gasdermin pores is activated by caspase-1, and these pores release the pro-inflammatory cytokines commonly found in cell death by pyroptosis [93,94]. Prolonged viral infection and obesity lower the immune response, which can result in cytokine storm and a substantial weakening of the immune system. The overexpression of IL-6 and chemokines CCL2, CCL4, CXCL8, CXCL9, and CXCL10 is associated with the pathogenicity of influenza viruses [95,96,97,98]. Chemokines CCL2, CXCL8, CXCL9, and CXCL10 are also related to mortality [97,99,100]. A(H1N1)pdm09 virus infection causes acute respiratory distress syndrome (ARDS) in obese people more often than in lean people. The development of viral ARDS in obese people is usually associated with a significantly higher level of IL-12 [99].

Influenza virus and SARS-CoV-2 differ in their ability to modulate immune response, which results in the production of different pro-inflammatory cytokines and chemokines. In contrast to influenza infection, infection with SARS-CoV-2 is less able to trigger the expression of IFNs. Despite the production of IFN-α2 being permanently abrogated, disease severity is related to the lower concentration of this IFN. IFN-β and IFN-λ are attenuated, and the level of IFN-γ is very low [101,102]. In patients positive with COVID-19, there are reduced concentrations of CD4^+^ and CD8^+^ T lymphocytes. In contrast, the level of helper Th17 cells is higher. SARS-CoV-2 upregulates the expression of five cytokines: IL-6, CCL2, CXCL1, CXCL5, and CXCL10 [17]. It was found that BMI did not correlate with the concentrations of IL-6, IL-8, IL-10, TNF-α, IFN-γ, CCL2, and CXCL10 in severely ill patients [103]. The massive release of TNF-α, IFN-γ, IL-1β, IL-8, CCL2, and CXCL10 in the acute phase of COVID-19 infection may lead to activation of the NLRP3 inflammasome and subsequent pyroptosis [104]. The course of COVID-19 may be affected by comorbidities associated with obese individuals. The cellular receptor ACE2 is secreted by mature adipocytes, and these are found in white and brown adipose tissue. Since obese individuals have more adipose tissue than lean individuals, they have an increased number of ACE2-expressing cells [105,106]. Since ACE2 is a functional receptor of SARS-CoV-2, the obesity-related overexpression of ACE2 may play a crucial role in increased susceptibility to SARS-CoV-2 and increased risk of acute respiratory failure. This process may be also responsible for the higher infectivity and dysfunction of other organs in obese individuals during SARS-CoV-2 infection [107].

Obese individuals are not more susceptible to SARS-CoV-2 infection. Cytokine storm and lipotoxicity are significantly associated with severe COVID-19 [108]. Under infection, SARS-CoV-2 binds to β cells, which results in an acute impairment of insulin secretion, acute hyperglycemic state and/or β-cell destruction, and the development of diabetes [109,110]. Newly diagnosed diabetes is responsible for the higher risk of mortality in patients with COVID-19 [110]. Hyperglycemia is responsible for the development of microangiopathy of alveolar capillaries and nonenzymatic glycation proteins in the lungs, and reduces the muco-ciliary clearance, so can enhance virus infection [111]. Systematic inflammation and insulin resistance produce oxidative stress and an inflammatory response in the pulmonary system, which can lead to lung abnormality [111,112]. It was shown that a high level of glucose affected influenza severity by increasing the pro-inflammatory response and damaging the epithelial–endothelial barrier in mice [113].

Monocytes infected with SARS-CoV-2 increase lipogenic transcription factors (peroxisome proliferator-activated receptor γ (PPARγ) and sterol regulatory element-binding protein 1 (SREBP-1)), increase the translation of proteins involved in lipid metabolism (fatty acid transporter CD36), and accumulate intracellular lipids [114]. Macrophages and monocytes infected with SARS-CoV-2 and influenza virus express the enzyme arachidonate 5-lipoxygenase (ALOX5), which is responsible for the production of arachidonic-derived mediators that increase leukotrienes, which can promote the production of pro-inflammatory adipokine [115,116]. Lipid droplets can serve as replication sites for the virus. Influenza virus promotes its replication by increasing the extracellular uptake of palmitic acid. Blocking CD36-dependent palmitate by using sulfo *N*-succinimidyl aleate can reduce viral replication [117]. Circulating palmitic acid in obese individuals binds TLR 2 and TLR 4 and activates their signaling pathways, resulting in increased inflammation [118]. The palmitoylation of viral membrane proteins promotes membrane fusion during SARS-CoV-2 and influenza virus entry into the cell [119,120].

The ACE2 receptor is also expressed on the surface of naïve and effector CD4^+^ and CD8^+^ T cells, which enables the SARS virus to infect them. Despite direct viral entry into lymphocytes being sporadically observed, the virus is not able to replicate in these cells [121,122]. In COVID-19 patients, the total amount of T cells is significantly lower in severely ill patients [123]. Lymphopenia was not observed in asymptomatic individuals or in symptomatic COVID-19 patients with pneumonia [124]. The high concentration of cytokines circulating in the blood of the patients with cytokine storm has a negative impact on T-cell proliferation and survival [125]. During viral infection, T cells reduce overreactive innate immune responses [126]. The loss of CD4^+^ and CD8^+^ T cells worsens the pathological inflammatory responses during SARS-CoV-2 infection. Additionally, excessively activated T cells can release cytotoxine granules and pro-inflammatory cytokines [127]. Persistent stimulation and long-term activation can induce both CD4^+^ and CD8^+^ T-cell exhaustion by expression of three exhaustion proteins: the CD28 family member programmed cell death marker 1 (PD-1), the receptor mucin domain-containing protein-3 (TIM-3), and the ITIM-bearing receptor NKG2A [125].

We already mentioned that mature adipocytes are present in white and brown adipose tissue. After infection with influenza virus, viral RNA and antigen-harboring cells were detected in the white tissue, and influenza infection induces browning features in preadipocytes [128]. Since the ACE2 receptor belongs to the renin-angiotensin system (RAS) family, SARS-CoV-2 modulates the RAS and energy metabolism in infected patients with obesity and diabetes mellitus [129]. The RAS enhances the activity of brown adipose tissue and induces the browning of white adipose tissue. Consequently, the increased energy is released in the form of heat instead of ATP synthesis. The affected metabolism of energy during viral infection in individuals with obesity leads to a worsening of their pathological conditions [130].

Adipose tissue and many residents of adipose tissue, such as adipocytes, endothelial cells, macrophages, and lymphocytes, can be infected with influenza virus and SARS-CoV-2 [121,131,132,133]. Both influenza virus and SARS-CoV-2 can be spread to adipose tissue by the blood or during local egress of the virus from infected organs [134,135]. After infection, specific inflammatory cytokines such as TNF-α, IL-1, and IL-6 are preactivated in adipose tissue, and these cytokines can amplify the immune response in infected lungs. The impaired balance between pro-inflammatory cytokines (TNFs and IL-1β) and their soluble cognate receptors can inhibit the cytokine effect and change the intensity of inflammation [136]. This aberrant cytokine activation leads to the development of cytokine storm. Adipose tissue can serve as an influenza virus and SARS-CoV-2 reservoir. Under infection, obesity is associated with disease severity, elevated virus titer in exhaled breath, and prolonged viral shedding. Moreover, the impaired IFN response allows the origination of new pathogenic variants of influenza virus with increased pathogenicity [137,138]. It is likely that obese individuals also pose a higher risk of the development of new pathogenic variants of SARS-CoV-2 virus.

## 8. Impact of Obesity on Vaccine Efficacy

The principle of vaccination is to prepare the immune system for more effective recognition and elimination of the infectious pathogens that they target. The body is able to elicit a faster immune response and effectively destroy a homologous pathogen, thereby preventing the onset of the disease, after repeated infection. It is well-known that an increased BMI is closely linked to a worse or lack of response to vaccination and may be associated with decreased antibody titers in adults and children. In studies of influenza vaccines, individuals with a high BMI had an initially higher-fold increase in IgG antibodies and hemagglutin inhibition titers than nonobese participants. There was a greater decline in antibody titers in obese participants 12 months post-vaccination [139,140]. Clinical data also suggested impaired immune responses, mainly adaptive T-cell response and decreased serological levels post-vaccination in the obese population [139,141,142]. In addition, Neidich et al. observed and confirmed that obese individuals had twice the risk of developing influenza post-vaccination compared to vaccinated individuals of normal weight and a BMI value range of 25 to 29.5 kg/m^2^ [143]. Notably, obese pregnant women may be more susceptible to respiratory infections, and they have a higher likelihood of developing various comorbidities (e.g., asthma, diabetes mellitus, hypertension, etc.) and problems with vaccination than normal-weight individuals [32]. Obese individuals exhibit poor B-cell responses and poor memory T-cell functions, which impair vaccine efficacy.

Today, a major concern is that COVID-19 vaccines will be less effective for obese people. The mRNA and vector vaccines are able to elicit virus neutralization antibodies as well as a T-cell immune response in obese individuals. The generation of memory T-cell populations is critical for an effective COVID-19 vaccine [144]. Unfortunately, it has already been shown that T-cell responses are impaired in individuals with obesity, which can suggest that future COVID-19 vaccines may be less effective in a population with a high prevalence of individuals with obesity. To date, no data strongly confirm the direct relation between COVID-19 vaccine effectiveness and obesity.

## 9. Conclusions

Obesity is associated with increased influenza and COVID-19 prevalence, severity, and mortality. The adipose tissue is now recognized as an endocrine organ that secretes inflammatory cytokines, adipokines, and chemokines. The different physiological and anatomical changes within the body of obese individuals compared to normal-weight individuals may contribute to the impaired immunity and modulation of antiviral immune responses. The adipose tissue contains a lower number of Th2 cells, M2 macrophages, and Treg cells; and increased amounts of pro-inflammatory cells such as CD8^+^ T cells and M1 macrophages. Adipose tissue resident cells such as neutrophils, dendritic cells, and mast cells release several pro-inflammatory factors and contribute to chronical inflammation. The dysregulation of fatty acid metabolism, a high level of glucose, insulin resistance, cellular hypertrophy, hypoxia, ER stress, and mitochondrial dysfunction lead to the significant alteration of the cellular architecture of the adipose tissue. This rearrangement facilitates a pro-inflammatory environment and maintains chronic inflammation. After infection with influenza virus and/or SARS-CoV-2, the abnormal metabolic environment and chronical inflammation have a negative impact on innate and adaptive immunity. The development of cytokine storm, and the increases in virus infectivity and virulence have a direct impact on disease severity and mortality. Adipose tissue can serve as a reservoir of infectious viruses, which can lead to elevated virus titers in exhaled breath and prolonged viral shedding. Moreover, the impaired IFN response allows the mutation of the virus to produce pathogenic variants of the virus with increased pathogenicity. BMI directly affects the efficiency of influenza vaccines. Data that confirm the influence of BMI on COVID-19 vaccine efficacy are still lacking. Therefore, vaccine trials and studies including BMI as a potential confounder must urgently be conducted to ensure vaccine effectiveness and protection.

## Supplementary Materials

The following are available online at www.mdpi.com/article/10.3390/v14010025/s1, Table S1: Summary of the COVID-19 meta-analyses. Table S2: Summary of the influenza meta-analyses.
