# Supplementary material for: Pandemics of the 21st Century: The Risk Factor for Obese People
Source: Viruses. 2021 Dec 23;14(1):25. doi: 10.3390/v14010025 (PMC8779521; doi:10.3390/v14010025)
Supplement: Supplementary file 1 [file viruses-14-00025-s001.zip › viruses-1461370-supplementary.pdf]

viruses

Review

Pandemics of the 21st Century: The Risk Factor for Obese People

Table S1. Summary of the covid-19 of meta-analyses.

Number of Studies and Patients	Country	Severe Outcome and Hospital. (OR)	ICU (OR)	IMV (OR)	Mortality (OR)	Publication
13 studies 7, 196 cases	USA, China, Italy, France	1.39; 95% CI = 1.21–1.60				[57]
33 studies 45, 650 cases	USA (18x), Italy (6x), China (1x), Spain (1x), Kuwait (1x), Mexico (3x), France (1x), Switzerland (1x), Greece (1x)	1.76; 95% CI = 1.21–2.56; *p* = 0.003	1.67; 95% CI = 1.26–2.21; *p* < 0.001	2.19; 95%CI = 1.56–3.07; *p* < 0.001	1.37; 95%CI = 1.06–1.75; *p* < 0.014	[58]
6 studies 2, 770 cases ICU; 5 studies 509 cases IMV	USA (4x), France (2x), Italy (1x), China (1x), Israel (1x), Singapore (1x)		1.21; CI = 1.002–1.46; I2 = 0.0%	2.05; 95%CI = 1.16–3.64; I2 = 34.86%		[66]
16 studies 6, 690 cases	USA (6x), Germany (1x), Switzerland (1x), Mexico (2x), France (2x), China (5x)	2.22; 95% CI = 1.40–3.53; *p* < 0.001	1.31; 95% CI = 0.87–1.99; *p* = 0.21			[59]
61 studies 270, 241 cases	not specified	3.13; 95% CI = 1.41–6.92; *p* = 0.005; I2 = 82.6	1.25; 95% CI = 0.99–1.58; *p* = 0.062; I2 =31.0%		1.36; 95%CI = 1.09–1.69; *p* = 0.006; I2 = 88.5	[60]
11 studies 9, 787 cases	USA (4x), China (3x), France (1x), Singapore (1x), Italy (1x), Mexico (1x)	2.07; 95% CI = 1.53–2.81; I2 = 70.9%			1.57; 95%CI = 0.85–2.19; I2 = 57%	[61]
11 studies 6, 081 cases	USA (6x), Spain (1x), Italy (2x), China (1x), Singapore (1x)	1.79; 95% CI = 1.52–2.11; *p* < 0.0001;I2 = 0%	1.86; 95% CI = 1.45–2.39; *p* < 0.0001, I2 = 0%	1.74; 95%CI = 1.39–2.17; *p* < 0.0001; I2 = 0%	1.05; 95%CI = 0.65–1.71; *p* = 0.84; I2 = 66.6%; not associated	[62]
9 studies 4, 444 cases	China (7x), USA (1x), France (1x)	pooled 2.31; 95% CI = 1.3–4.12				[63]
10 studies 10, 233 cases	USA (6x), China (3x), Singapore (1x)	1.88; 95% CI = 1.25–2.80; *p* = 0.002; with 86% heterogeneity between studies *p* < 0.00001				[64]
17 studies 543, 399 cases	Mexico (1x), USA (10x), France (1x), UK (1x), Italy (2x), China (1x), Multiple countries (1x)				1.42; 95%CI = 1.24–1.63; *p* < 0.001	[67]
16 studies 109, 881 cases	China (3x), Kuwait (1x), USA (7x), Mexico (2x), Italy (2x), France (1x)	2.35; 95% CI = 1.64–3.38; *p* < 0.001			2.68; 95%CI = 1.65–4.37; *p* < 0.001	[65]

OR = odds ratio; CI = Confidence interval; *p*-value= probability value; I2= I-squared statistic of heterogeneity; ICU= Intensive Care Unit; IMV= Invasive Mechanical Ventilation.

Table S2. Summary of the influenza meta-analyses.

Number of Studies and Patients	Country	Hospit. ICU (OR)	Mortality (OR)	Hospit. ICU (OR)	Mortality (OR)	Publication
		obese (30.0 ≤ BMI < 35.0)		morbidly obese (BMI ≥ 35.0)		
Pandemic Influenza						
6 studies 3, 059 cases	Canada (1x), USA (2x), Ireland (1x), France (1x), Netherlands (1x)	2.14; 95% CI= 0.92–4.99; *p* < 0.07		2.01; 95% CI= 1.29–3.14; *p* < 0.002		[76]
183 studies 534, 911 cases	not specified	3.44; 95% CI= 2.14–5.54; I2 = 71%	2.74; 95% CI= 1.56–4.80; I2 = 92%			[77]
22 studies 25, 189 cases	Asia region	1.67; 95% CI= 1.13–2.47	1.81; 95% CI= 1.23–2.65			[78]
35 studies 4, 023, 895 cases	USA (12x), Spain (4x), China (6x), Canada (3x), UK (2x), Multiple countries (1x)	1.56; 95% CI= 1.28–2.04; I2: 82.3%	1.99; 95% CI= 1.15–3.46; I2= 82.7%	3.08; 95% CI= 1.43–6.62; I2= 92%	1.40; 95% CI= 1.10–1.79; I2: 81.7%	[61]

OR = odds ratio; CI = Confidence interval; *p*-value= probability value; I2= I-squared statistic of heterogeneity; ICU= Intensive Care Unit.
